# Comparison between two analytic strategies to detect linkage to obesity with genetically determined age of onset: the Framingham Heart Study

**DOI:** 10.1186/1471-2156-4-S1-S90

**Published:** 2003-12-31

**Authors:** Corinne D Engelman, Heather L Brady, Anna E Baron, Jill M Norris

**Affiliations:** 1 Department of Preventive Medicine and Biometrics, University of Colorado Health Sciences Center, Denver, Colorado, USA

## Abstract

**Background:**

Genes have been found to influence the age of onset of several diseases and traits. The occurrence of many chronic diseases, obesity included, appears to be strongly age-dependent. However, an analysis of potential age of onset genes for obesity has yet to be reported. There are at least two analytic methods for determining an age of onset gene. The first is to consider a person affected if they possess the trait before a certain age (an early age of onset phenotype). The second is to define the phenotype based on the residual from a survival analysis.

**Results:**

No regions provided evidence for linkage at the more stringent level of p < 0.001. However, five regions showed consistent suggestive evidence for linkage (one marker with p < 0.01 and a second contiguous marker at p < 0.05). These regions were chromosome 1 (280–294 cM) and chromosome 16 (56–64 cM) for overweight using the survival analysis residual method and chromosome 13 (102–122 cM), chromosome 17 (127–138 cM), and chromosome 19 (23–47 cM) for obese before age 35.

**Conclusion:**

Only one region (chromosome 19 at 23–47 cM) showed somewhat consistent results between the two analytic methods. Potential reasons for inconsistent results between the two methods, as well as their strengths and weaknesses, are discussed. The use of both methods together to explore the genetics of the age of onset of a trait may prove to be beneficial in determining a gene that is linked only to an early age of onset phenotype versus one that determines age of onset through all age groups.

## Background

In the United States, the prevalence of obesity has increased dramatically in the past several decades. Results from the Behavioral Risk Factor Surveillance System indicated that obesity prevalence has increased by more than 50% in adults between 1991–1999 [1,2]. According to the definition of the World Health Organization (WHO), approximately 6–10% of the population in Westernized countries is considered obese [3]. Epidemiological studies have shown that between 30–70% of the variation in body weight may be attributable to genetic factors [4]. Obesity related traits, such as body mass index (BMI), are influenced by both genetic and environmental factors. BMI is an easy, reliable, accurate measurement and is highly correlated (0.8–0.9) with total fat mass in adults [5]. A number of studies have estimated the heritability of BMI to be 40%-55% [4,6], but estimates greater than 80% have been found in twin studies.

Mendelian types of obesity have been identified [7], although the genes that confer susceptibility to the common form of obesity are largely unknown. Segregation analyses have provided evidence for a recessive major gene that accounts for 20–40% of the variance with an additional 34–42% of the variance attributed to a multifactorial component [8,9]. Additional segregation analyses using two-locus models in independent studies have revealed evidence for two recessive loci that together account for approximately 64–68% of the variance [10,11]. In addition, it appears as though some genotypes may interact with age and sex [12].

The occurrence of many chronic diseases, obesity included, appears to be strongly age-dependent and it may be necessary to account for this in linkage studies designed to detect these genes. A genome-wide linkage analysis in the Framingham Heart Study population offers an opportunity to examine both age of onset and ultimate susceptibility to overweight and obesity, as measured by body mass index (BMI).

Specifically, this can be addressed by applying two methods for analyzing the age-dependent genetic factors that may impact overweight status and/or obesity. The first method entails restricting the analysis to those with an age of onset prior to some arbitrary threshold. The second strategy is a modification of nonparametric analyses such that it accounts for age-specific occurrence. This latter method involves defining the trait as a "residual" from a survival analysis model [13,14]. These two methods are useful because each will explore different questions; that is, the restriction analysis will address an early age of onset phenotype of obesity/overweight, whereas defining a "residual" examines the age of onset of obesity/overweight throughout the life-span.

The objectives of this study are two-fold: 1) To test for linkage to an early age of onset phenotype of obesity/overweight (i.e., BMI) using restriction analysis. This analysis will reduce age-related heterogeneity in which young affected individuals possess a susceptibility gene for early age of onset obesity/overweight that is different from the disease gene possessed by those who develop the disease later in life. 2) To test for linkage to the variation of age of onset of obesity/overweight throughout the life-span by defining the phenotype (i.e., BMI) as a "residual" in a survival analysis model. In this case, when the genetic effect is on the variation in age of onset, the disease allele influences the age at which the person develops the disorder.

## Methods

The Framingham Heart Study data set for the Genetic Analysis Workshop 13 was utilized to conduct a genome-wide linkage analysis on 1702 individuals in 330 pedigrees. All analyses were performed on sibling pairs using SIBPAL (S.A.G.E. version 4.2). The GENIBD program (S.A.G.E. version 4.2) was used for generating single-point identity-by-descent (IBD) sharing distributions. All of the default options were used in the GENIBD and SIBPAL analyses.

In the present study, we used BMI as the measure of obesity/overweight. BMI was calculated as weight (in kg) divided by the square of the height (in meters). The mean height was used because height measurements at each clinic visit may have varied for each subject.

To examine the phenotypic heterogeneity of overweight and obesity in which a genetic effect is involved in the age of onset, the analyses were restricted to those with an age of onset < 35 years. This age of onset was chosen in order to maximize sample size while minimizing the behavioral effects of decreasing leisure time physical activity that frequently occur in the 30 s and 40 s (preliminary descriptive statistics [data not shown] indicated that the minimum age of onset to provide enough "affected" individuals was about 35 years). A BMI of > 27 was defined as overweight and > 30 was defined as obese. Age of onset was the age at which an individual first met the BMI criteria or, if they met the BMI criteria at the start of the study, their age at the baseline visit. The restricted analysis was performed for the phenotype of overweight and also for the phenotype of obesity using the test of mean allele sharing for binary traits in concordantly affected sibling pairs (in SIBPAL).

The survival analysis "residual" method uses PROC LIFETEST (S.A.S. version 8.1) to obtain the cohort and sex-specific cumulative incidence at the age a person was first affected or, if unaffected, the age at the last visit. This cumulative incidence was subtracted from the affection status (0 for unaffected and 1 for affected) to obtain the "residual" (the adjusted affection status). This approach was performed on the phenotypes of overweight and of obesity using the traditional Haseman-Elston regression model (SIBPAL).

## Results

A total of 94 sibling pairs concordantly overweight before age 35 and 28 sibling pairs concordantly obese before age 35 were included in the restricted analysis and 1500 sibling pairs were in both survival analyses. The mean BMI at baseline for Cohort 1 (the original Framingham cohort) was 27 (mean baseline age of 42 years) and for Cohort 2 (the offspring of the original Framingham cohort) was 26 (mean baseline age of 33 years). Selected results from single-point linkage analysis for the phenotypes of overweight and obesity are shown in Table 1. Contiguous regions of chromosomes are indicated by an partial lines. Only markers that achieved a significance level of *p *< 0.01 or markers with *p *< 0.05 that were clustered together were reported.

No regions provided evidence for linkage at the more stringent level of *p *< 0.001. The results for the phenotype of *overweight before age 35 *provide suggestive evidence for linkage to regions on chromosome 6 (182 cM), with weak evidence for linkage to chromosome 1 (120–125 cM), chromosome 3 (77–98 cM), and chromosome 20 (44–62 cM). The results for the phenotype of *overweight using survival analysis residual *provide suggestive evidence for linkage to chromosome 1 (280–294 cM) and chromosome 16 (25–64 and 95 cM), with weak evidence for linkage to chromosome 5 (63–92 cM). The results for the phenotype of *obese before age 35 *provide suggestive evidence for linkage to chromosome 7 (55 cM), chromosome 13 (102–122 cM), chromosome 17 (127–138 cM), and chromosome 19 (23–47 and 112 cM), with weak evidence for linkage on chromosome 20 (90–108 cM). The results for the phenotype of *obese using survival analysis residual *provide suggestive evidence for linkage to chromosome 7 (32–45 cM) with weak evidence for linkage on chromosome 5 (125–142 cM).

## Discussion

The affected relative/sib pair approach has been widely used in linkage analysis. One of the major advantages of this approach is that it is not affected by age-related penetrance (variable age of onset) because all individuals in the analysis are affected. However, there are at least two situations in which this approach alone is not sufficient. First, phenocopies that appear to be identical to a genetic trait but are caused by nongenetic factors may decrease the power of an affected relative/sib-pair analysis, making it less than ideal. Second, phenotypic heterogeneity (where different genes cause different ages of onset) may lead to two affected relative/sibling pairs having different ages of onset due to different genes and, thus, a decrease in the power to detect linkage. Therefore, it is of interest to find an analytic approach that addresses these issues. Restricting the analysis to the phenotype of early age of onset of obesity/overweight (<35 years) attempts to reduce the phenotypic heterogeneity due to differences in age of onset. Another aim of this analytic approach is to "weed out" phenocopies of obesity/overweight that may occur later in life and may be caused by other genetic or environmental factors. The survival analysis residual method attempts to detect a genetic effect on the age of onset of obesity/overweight. This method accounts for cohort, sex, and age-specific risk for obesity/overweight in the sample population, by determining the cumulative incidence in the sample for a particular cohort, sex, and age. For example, the value of the residual (the adjusted affection status) would be 0.8 for an affected person when the cohort, sex- and age-specific cumulative incidence was 0.2 (fewer people had become obese/overweight in their group) and 0.2 if the cumulative incidence was 0.8 (more people had become obese/overweight in their group). The person with the residual of 0.8 could potentially have a genetic risk factor predisposing them to an earlier age of onset than the person with the residual of 0.2. The restriction method would be expected to perform well in the case of an early age of onset phenotype that is etiologically distinct from later age of onset phenotypes. The survival analysis residual method would be expected to be more robust when there is a genetic effect on the variation in the age of onset throughout the life-span. In this case, loss of information would occur by using an age of onset cut-off to restrict the analysis. A strong genetic effect on age of onset throughout the life-span (including ages less than the cut-off age of the restricted analysis) may be expected to produce consistent results between both analytic methods.

In our genome-wide linkage analysis, five regions showed consistent suggestive evidence for linkage (one marker with p < 0.01 and a second contiguous marker at p < 0.05). These regions were chromosome 1 (280–294 cM) and chromosome 16 (56–64 cM) for overweight using the survival analysis residual method and chromosome 13 (102–122 cM), chromosome 17 (127–138 cM), and chromosome 19 (23–47 cM) for obese before age 35.

The results on chromosomes 1 and 16 for overweight using the survival analysis residual method were not replicated in the affected sibling pairs with age of onset of overweight before 35. This region may be responsible for a genetic effect on age of onset of overweight in the middle and later years of life when, in general, a more sedentary lifestyle has been adopted. This may be the case if the region was involved in a gene × environment interaction in which a sedentary lifestyle in addition to the gene was necessary to result in moderate weight gain. Another explanation is that the number of affected sibling pairs was too small to detect linkage, whereas using all the sibling pairs in the survival analysis residual method (a continuous trait) resulted in more power to detect linkage.

The results on chromosomes 13 and 17 for obese before age 35 were not replicated by the survival analysis residual method. This may indicate that chromosomes 13 and 17 are linked with a distinct phenotype of obesity in which the age of onset is before 35 years of age. These regions may even be responsible for a phenotype of childhood or adolescent obesity, although we did not have enough people in these age groups to test this hypothesis.

Another explanation for the inconsistency of the results on chromosomes 13 and 17 could be strong environmental determinants of shifting from normal weight or overweight to obese in the middle and later years of life (age of onset ≥ 35). If a gene(s) plays a role in the age at which one becomes obese, it may be more easily detected at a younger age in which people tend to be more physically active (the environmental exposure of a sedentary lifestyle is absent). As many people become more sedentary in their 30s and beyond, weight gain may occur due to excess energy intake even in the absence of genetic factors, making it more difficult to detect a genetic effect. The stratified analysis of people who were obese at a younger age would not be as affected by this shift in environmental exposure. This approach would perform the linkage analysis on a more homogeneous population of affected individuals. Conversely, although the survival analysis residual method accounts for excess energy intake with increasing age in the population through adjustment of the affection status by the cumulative incidence, all individuals are included in the analysis, potentially creating a more heterogeneous sample with respect to etiology of the phenotype. Hypothetically, a discordant sibling pair in which one sibling became affected at age 40 entirely due to excess energy intake (assume no genetic susceptibility) while the other sibling was unaffected at age 40 and did not have an excess energy intake would tend to "wash out" a true genetic effect on the phenotype in another sibling pair.

There was one region (chromosomes 19 at 22 cM) in which both analytic methods detected evidence for linkage to the phenotype of obesity. Consistency of results from the two analytic methods may have occurred due to a genetic effect on age of onset throughout the lifespan including ages < 35.

Overall, both analytic methods have strengths and weaknesses and the use of both methods together to explore the genetics of the age of onset of a trait may prove to be beneficial in determining a gene that is linked only to an early age of onset phenotype versus one that determines age of onset through all age groups. The survival analysis residual method allows for the entire sample to be analyzed, whereas the stratification method limits one to a smaller sample size, which reduces one's power. Additionally, stratification may lose information from siblings who are not affected at the time of the visit, but may later become affected, whereas the survival analysis residual method uses all siblings and adjusts their affection status by the cumulative incidence of the trait. However, stratification by age may be better able to detect linkage if there is a strong environmental component in ages greater than the stratification cutoff age.

## Figures and Tables

**Table 1:** Summary of significant linkage findings for overweight and obesity using two analytic approaches.

			***p*-Values**			
			
			**Overweight**		**Obese**	
			
**Chromosome**	**Marker**	**Distance (Order on Chromosome)**	**Age of** ** Onset <35**	**Survival Analysis Residual**	**Age of** ** Onset <35**	**Survival Analysis Residual**
1	GATA109	119.79 (12)	0.04^A^	0.93	0.05	0.60
1	GATA6A05	125.17 (13)	0.02^A^	0.44	0.05	0.49
		
1	203yg9	280.12 (30)	0.50	0.02^A^	0.38	0.25
1	GATA4A09	294.45 (31)	0.92	0.007^B^	0.50	0.63
		
3	ATA10H11	76.97 (9)	0.04^A^	0.59	0.02^A^	0.64
3	GATA148E04	98.15 (11)	0.03^A^	0.76	0.29	0.32
		
5	GATA21D04	63.39 (11)	0.88	0.04^A^	0.65	0.84
5	GATA52A12	92.30 (13)	0.70	0.02^A^	0.73	0.67
		
5	ATA4D10	125.22 (17)	0.22	0.14	0.11	0.03^A^
5	GATA62A04	141.70 (19)	0.64	0.22	0.66	0.047^A^
6	242zg5	181.68 (20)	0.003^B^	0.72	0.19	0.18
		
7	GATA137H02	31.97 (3)	0.92	0.19	0.61	0.003^B^
7	GATA41G07	36.30 (4)	0.92	0.04^A^	0.88	0.19
7	GGAA3F06	45.24 (5)	0.92	0.27	0.36	0.03^A^
7	GATA13G11	54.60 (6)	0.76	0.31	0.003^B^	0.15
		
13	261yg5	102.47 (11)	0.36	0.31	0.02^A^	0.85
13	309va9	122.40 (12)	0.11	0.31	0.001^B^	0.27
		
16	GATA71H05	56.14 (5)	0.42	0.02^A^	0.10	0.22
16	GGAA3G05	63.74 (6)	0.80	0.0098^B^	0.28	0.08
16	GATA81D12	95.47 (10)	0.80	0.003^B^	0.42	0.48
		
17	044xg3	127.48 (15)	0.15	0.37	0.006^B^	0.92
17	217yd10	138.03 (16)	0.11	0.18	0.01^A^	0.92
		
19	GATA21G05	23.03 (2)	0.64	0.72	0.003^B^	0.06
19	GATA66B04	46.82 (4)	0.66	0.31	0.04^A^	0.82
19	Mfd238	111.74 (11)	0.10	0.39	0.001^B^	0.41
		
20	GGAA7E02	44.46 (5)	0.03^A^	0.60	0.04^A^	0.14
20	GATA42A03	61.63 (7)	0.04^A^	0.56	0.59	0.39
		
20	GATA45B10	89.70 (10)	0.46	0.29	0.04^A^	0.24
20	046xf6	108.32 (11)	0.39	0.38	0.03^A^	0.48

^A^*p *< 0.05 ^B^*p *< 0.01 Sections divided by partial lines indicate contiguous markers.
